# Supplementary figures and images for: CYP2C11 played a significant role in down-regulating rat blood pressure under the challenge of a high-salt diet
Source: PeerJ. 2019 Apr 23;7:e6807. doi: 10.7717/peerj.6807 (PMC6485204; doi:10.7717/peerj.6807)

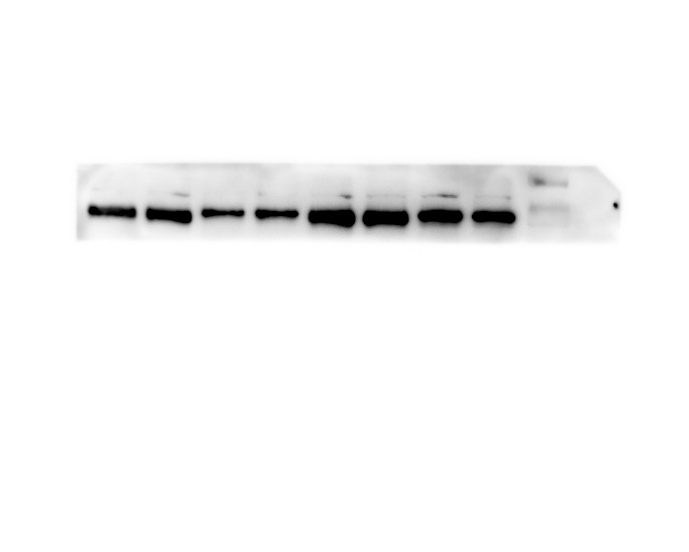

Supplement: Supplemental Information 2 — All uncropped and modified figures of rat western blots on a high-salt diet and a normal diet. [file peerj-07-6807-s002.zip › full-length uncropped blots (Figure 2)/CYP2J2-high-salt.tif]

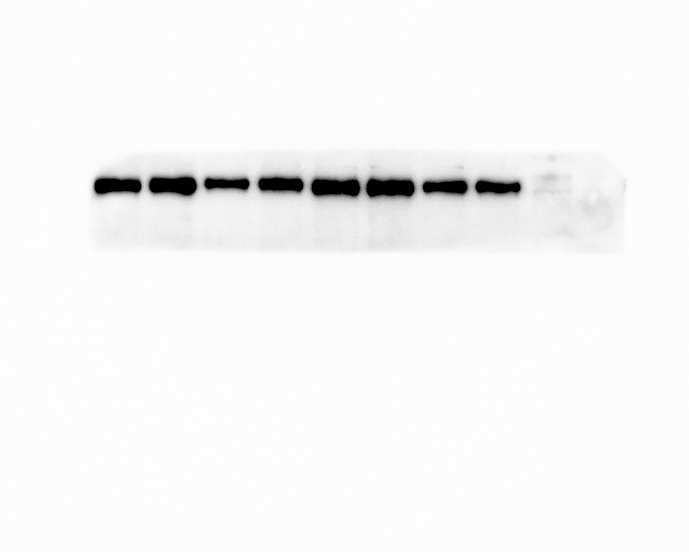

Supplement: Supplemental Information 2 — All uncropped and modified figures of rat western blots on a high-salt diet and a normal diet. [file peerj-07-6807-s002.zip › full-length uncropped blots (Figure 2)/CYP2J2-normal salt.tif]

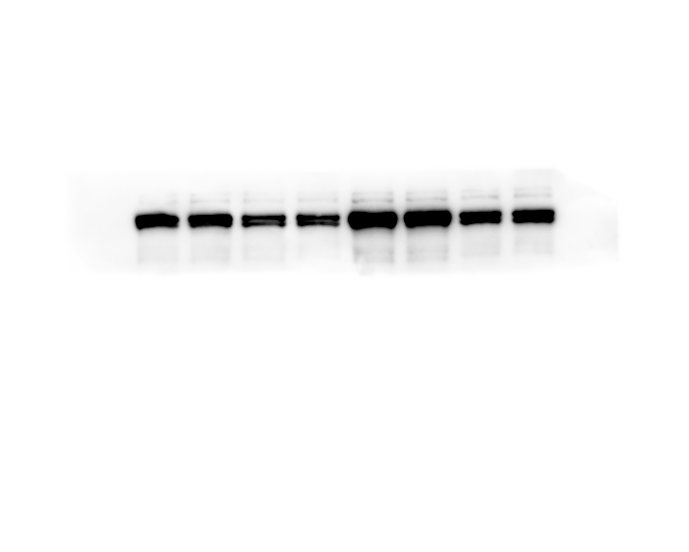

Supplement: Supplemental Information 2 — All uncropped and modified figures of rat western blots on a high-salt diet and a normal diet. [file peerj-07-6807-s002.zip › full-length uncropped blots (Figure 2)/CYP4A-high-salt.tif]

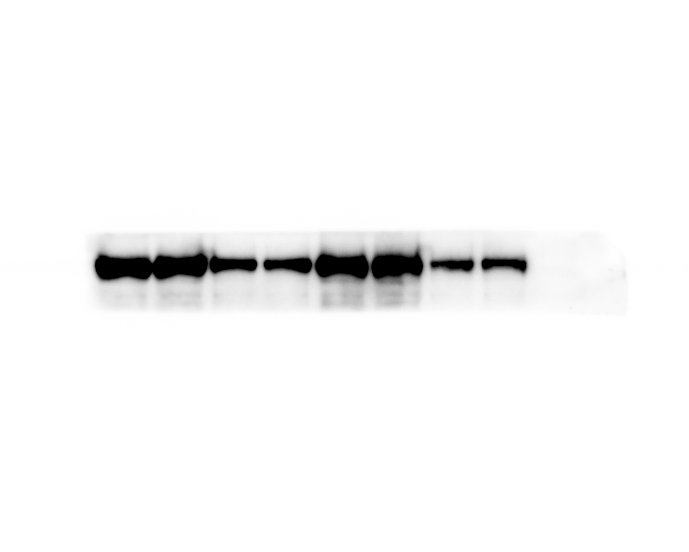

Supplement: Supplemental Information 2 — All uncropped and modified figures of rat western blots on a high-salt diet and a normal diet. [file peerj-07-6807-s002.zip › full-length uncropped blots (Figure 2)/CYP4A-normal salt.tif]
